# A score to quantify coronary plaque vulnerability in high-risk patients with type 2 diabetes: an optical coherence tomography study

**DOI:** 10.1186/s12933-014-0117-8

**Published:** 2014-08-21

**Authors:** Mathias Burgmaier, Martin Hellmich, Nikolaus Marx, Sebastian Reith

**Affiliations:** Department of Cardiology, Medical Clinic I, University Hospital of the RWTH Aachen, Pauwelsstr 30, D-52074 Aachen, Germany; Institute of Medical Statistics, Informatics and Epidemiology, University of Cologne, Cologne, D-50924 Germany

**Keywords:** Optical coherence tomography, Plaque vulnerability, Type 2 diabetes mellitus, Acute coronary syndrome

## Abstract

**Background:**

Patients with type 2 diabetes are at a high risk for acute cardiovascular events, which usually arise from the rupture of a vulnerable coronary lesion characterized by specific morphological plaque features. Thus, the identification of vulnerable plaques is of utmost clinical importance in patients with type 2 diabetes. However, there is currently no scoring system available to identify vulnerable lesions based on plaque characteristics. Thus, we aimed to characterize the diagnostic value of optical coherence tomography (OCT) - derived lesion characteristics to quantify plaque vulnerability both as individual parameters and when combined to a score in patients with type 2 diabetes.

**Methods:**

OCT was performed in the coronary culprit lesions of 112 patients with type 2 diabetes. The score, which quantifies plaque vulnerability, was defined as the predicted probability that a lesion is the cause for an acute coronary syndrome (ACS) (vs. stable angina (SAP)) based on its specific plaque morphology.

**Results:**

Multivariable logistic regression analysis demonstrated that plaque vulnerability was independently predicted by the minimal fibrous cap thickness overlying a lesion’s lipid core (odds ratio (OR) per 10 μm 0.478, p = 0.002), the medium lipid arc (OR per 90° 13.997, p < 0.001), the presence of macrophages (OR 4.797, p = 0.015) and the lipid plaque length (OR 1.290, p = 0.098).

Receiver-operating-characteristics (ROC) analyses demonstrated that these parameters combined to a score demonstrate an excellent diagnostic efficiency to identify culprit lesions of patients with ACS (vs. SAP, AUC 0.90, 95% CI 0.84-0.96).

**Conclusion:**

This is the first study to present a score to quantify lesion vulnerability in patients with type 2 diabetes. This score may be a valuable adjunct in decision-making and useful in guiding coronary interventions.

## Introduction

Patients with type 2 diabetes are at a high risk for acute cardiovascular events. Acute coronary syndromes (ACS) usually arise from the rupture of vulnerable coronary lesions, which in turn may result in thrombus formation and the occlusion of a coronary vessel [1]. Recently, several characteristics of vulnerable plaques have been suggested in both histopathological as well as clinical studies [2,3]. These include a thin fibrous cap overlying a large lipid core, plaque inflammation and the presence of microchannels [2,4,5]. However, currently there is no scoring system available to quantify plaque vulnerability, which could be used to identify lesions at risk to cause ACS based on individual plaque characteristics. Furthermore, the relative contribution of these morphological markers to a vulnerable plaque is incompletely understood.

With the emerging use of intravascular imaging modalities such as virtual histology intravascular ultrasound (IVUS) and optical coherence tomography (OCT) it is possible to identify these vulnerable plaque features [6,7]. Compared to IVUS, OCT offers a resolution ~10 times higher and allows for the first time the visualization of plaque inflammation *in vivo* [8]. However, as scoring systems are missing, the clinical consequence following the recognition of one or more of these vulnerable plaque features by the interventional cardiologist remains uncertain.

Identifying and treating vulnerable lesions is a major goal particularly in patients with type 2 diabetes, who are at a high risk for cardiovascular events [9]. Thus, the identification of vulnerable plaques using an OCT-based scoring system may have an important impact on choosing the optimal therapeutic strategy particularly in this high-risk population.

We have recently demonstrated a lower fibrous cap thickness (FCT), a larger necrotic lipid core and pronounced plaque inflammation in culprit lesions of diabetic patients with ACS compared to lesions in patients with stable angina pectoris (SAP) [5]. Furthermore, recently a prospective study has demonstrated, that ACS usually arise from the rupture of low grade stenotic lesions, which are vulnerable [10]. Taken together, the recognition and treatment of vulnerable plaques may be an interesting concept and important particularly in high-risk patients with type 2 diabetes.

The aim of this study was to characterize the diagnostic value of OCT-derived lesion characteristics to predict plaque vulnerability both as individual parameters and when combined to a score.

## Methods

### Study population

112 patients with type 2 diabetes undergoing coronary angiography due to ACS or SAP and subsequent percutaneous coronary intervention of the coronary culprit lesion were enrolled in the present investigation at the Department of Cardiology, University Hospital of the RWTH Aachen, Germany between June 2011 and February 2014. OCT and quantitative coronary angiography (QCA) analysis, laboratory testing and clinical history taking were performed in all patients as previously described [11,12]. A subset of the diabetic cohort was part of a former analysis [5].

In the SAP-group lesion identification was based on the coronary angiogram with an at least intermediate grade stenosis (>40% on QCA) suitable for coronary intervention and confirmed by additional information from electrocardiographic, echocardiographic or MRT-derived stress testing and/or fractional flow reserve measurements. In patients with ACS the target lesion was identified due to the evidence of plaque rupture with or without local thrombus, electrocardiographic findings and/or echocardiographic wall motion abnormalities. In case of a TIMI flow grade ≤ II within the target vessel we performed aspiration thrombectomy to restore TIMI flow III before intracoronary OCT image acquisition [5].

Further inclusion criteria were type 2 diabetes, age >30 years and written informed consent to the study protocol. Type 2 diabetes was based on clinical history of diabetes mellitus, ongoing insulin/oral antidiabetic therapy and/or an HbA_1C_-level exceeding 6.5% [5].

Exclusion criteria were left main coronary artery stenosis, bypass graft lesions, cardiogenic shock, hemodynamic or rhythmic instability, acute or chronic renal insufficiency (serum creatinine level >1.5 mg/dl) and pregnancy. We furthermore excluded severely tortuous and calcified vessels, not allowing the safe advancement of the OCT catheter and the necessity to predilate a target lesion prior to OCT in order to achieve TIMI flow grade III [5].

The study was approved by the local Ethics Committee and is in accordance with the Declaration of Helsinki on ethical principles for medical research involving human subjects.

### OCT image acquisition and analysis

For image acquisition of the target lesion we used the Frequency-Domain-OCT C7XR system and the DragonFly catheter (St. Jude Medical Systems, LightLab Imaging Inc., Westford, Massachusetts, USA) with an automated pull back device at a rate of 20 mm/s as previously described [12]. We achieved blood clearance by the non-occlusion OCT-technique with injection of isoosmolar contrast (Iodixanol, GE Healthcare, USA).

Quantitative intraluminal dimensions and plaque morphology were assessed as previously described [5,11,12].

Subsequent offline and pull-back analysis was performed by two independent observers throughout the entire lesion frame by frame in 0.2 mm intervals, using the proprietary software provided by LightLab Imaging and in adaptation to the published consensus for quantitative and qualitative assessment [5,7,13]. In case of discordant results, a consensus measurement was taken.

### Statistical analysis

Statistical analyses were performed with SPSS software (IBM Corp., Armonk, NY, USA). Categorical variables were summarized as count (percentage), continuous variables as mean ± standard deviation. To investigate the diagnostic value of morphologic plaque features to predict that the lesion is the cause for an ACS, univariable logistic regression analysis was performed. Plaque parameters which showed a p-value (Wald statistics) below 0.05 were also studied in a multivariable logistic regression analysis with consecutive backward selection for variables which had a p-value below 0.10. In case of a non-lipid plaque, parameters which were not defined for non-lipid plaques such as FCT, lipid arc, and lipid plaque length were set to 0. This is both consistent with the sign of association of each of these variables with the dependent variable (i.e. ACS) and may formally be derived from introducing interactions of these variables with the type of plaque.

The score was calculated based on the multiple regression equation and represents the predicted probability of a lesion to be the cause for an ACS (vs. SAP) based on its specific plaque morphology. Test results are reported as p-value (p), odds ratio (OR) and corresponding 95% limits of confidence (CI). Sensitivity, specificity, positive predictive value (PPV), negative predictive value (NPV) and optimal cut-off-values were calculated from the receiver operating characteristic (ROC) curve to identify culprit lesions of patients with ACS. Values with the highest Youden-index (sensitivity + specificity - 1) were identified as optimal cut-off-values. A classification of the diagnostic efficiency according to the values of the area under the curve (AUC) was used as described elsewhere [14]. A p-value < 0.05 was considered to indicate statistical significance.

## Results

### Clinical and lesion characteristics

A total of 112 patients were enrolled in the study and categorized into a group of 43 patients with ACS and a group of 69 patients with SAP. The culprit lesion was examined by OCT in all patients without any peri- or postprocedural complications. Clinical and lesion characteristics are summarized in Table 1.

**Table 1:** **Univariable logistic regression analysis for the lesion to be the cause of an ACS**

**Parameter**	**ACS (n = 43)**	**SAP (n = 69)**	**OR**	**95% CI**	**p-value**
**Clinical:**					
Age (years)	68.1 ± 9.9	69.6 ± 7.4	0.98	(0.94 - 1.02)	ns
Male (n, %)	28 (65.1)	47 (68.1)	0.87	(0.39 - 1.96)	ns
BMI (kg/m^2^)	29.7 ± 5.8	31.5 ± 4.8	0.93	(0.86 - 1.01)	0.081
Hypertension (n, %)	35 (81.4)	60 (87.0)	0.67	(0.23 - 1.86)	ns
MAP (mmHg)	93.7 ± 11.5	96.3 ± 13.0	0.98	(0.95 - 1.01)	ns
Dyslipidemia (n, %)	26 (60.5)	47 (68.1)	0.72	(0.32 - 1.58)	ns
Smoking (n, %)	13 (30.2)	11 (15.9)	2.29	(0.91 - 5.71)	0.077
Family history (n, %)	14 (32.6)	31 (44.9)	0.59	(0.27 - 1.31)	ns
Diabetes (years)	12.3 ± 10.5	11.0 ± 9.8	1.01	(0.98 - 1.05)	ns
Hb_A1C_ (%)	7.78 ± 1.73	7.19 ± 1.45	1.27	(0.98 - 1.65)	0.074
Fasting glukose (mg/dl)	174.8 ± 70.6	164.4 ± 46.7	1.00	(1.00 - 1.01)	ns
Total chol (mg/dl)	191.1 ± 43.0	189.8 ± 43.8	1.00	(0.99 - 1.01)	ns
LDL-chol (mg/dl)	116.7 ± 36.1	117.0 ± 35.7	1.00	(0.99 - 1.01)	ns
HDL-chol (mg/dl)	43.9 ± 14.1	43.9 ± 10.5	1.00	(0.97 - 1.03)	ns
Triglyceride (mg/dl)	166.1 ± 92.3	187.1 ± 103.6	1.00	(0.99 - 1.00)	ns
CRP (mg/dl)	20.4 ± 30.3	9.1 ± 10.7	1.03	(1.01 - 1.06)	0.016
**OCT-derived parameters**					
MLA (mm^2^)	1.39 ± 0.76	1.42 ± 0.66	0.94	(0.54 - 1.63)	ns
MLD (mm)	1.06 ± 0.26	1.13 ± 0.25	0.30	(0.06 - 1.58)	ns
Ref LA (mm^2^)	6.81 ± 2.36	6.51 ± 2.07	1.06	(0.89 - 1.27)	ns
Stenosis length (mm)	9.13 ± 4.22	7.98 ± 5.39	1.05	(0.97 - 1.13)	ns
Area stenosis (%)	79.49 ± 7.14	77.34 ± 8.62	1.03	(0.99 - 1.09)	ns
Calcified Pl. (n, %)	26 (60.5)	48 (69.6)	0.67	(0.30 - 1.49)	ns
Fibrous Pl. (n, %)	33 (76.7)	62 (89.9)	0.37	(0.13 - 1.07)	0.066
Lipid-rich Pl. (n, %)	36 (83.7)	29 (42.0)	7.09	(2.77 - 18.16)	<0.001
TCFA n (%)	32 (88.9)	10 (34.5)	15.20	(4.18 - 55.28)	<0.001
Non-TCFA (n, %)	3 (8.3)	19 (65.5)	0.05	(0.01 - 0.20)	<0.001
Min FCT (10 μm)	5.25 ± 0.94	8.00 ± 2.44	0.39	(0.24 - 0.64)	<0.001
Mean FCT (10 μm)	9.75 ± 1.71	12.14 ± 2.44	0.57	(0.41 - 0.79)	0.001
Lipid arc (90°)	1.98 ± 0.39	1.39 ± 0.47	18.08	(4.50 - 72.60)	<0.001
LPL (mm)	6.34 ± 2.04	3.58 ± 2.14	1.80	(1.34 - 2.40)	<0.001
LVI (per 1000)	11.05 ± 3.39	4.81 ± 3.66	1.53	(1.26 - 1.86)	<0.001
Macrophages (n, %)	35 (81.4)	23 (33.3)	8.75	(3.50 - 21.89)	<0.001
MC (n %)	25 (58.1)	26 (37.7)	2.30	(1.06 - 5.00)	0.036

The data are presented as mean ± SD or n (%) as well as the odds ratio for the vulnerable plaque with the 95% confidence interval (95% CI).

Abbreviations: *ACS* acute coronary syndrome, *SAP* stable angina pectoris, *BMI* body mass index, *MAP* mean arterial pressure, *Fast*. Fasting, *LDL* low density lipoprotein, *HDL* high density lipoprotein, *CRP* C-reactive protein, *OCT* optical coherence tomography, *MLA* minimal lumen area, *MLD* minimal lumen diameter, Ref. *LA* reference luminal area, *Pl*. plaque, *TCFA* thin-capped fibroatheroma, *FCT* fibrous cap thickness, *LPL* lipid plaque length, *LVI* lipid volume index, *MC* microchannel.

### Risk factors for plaque vulnerability

**Figure 1:**
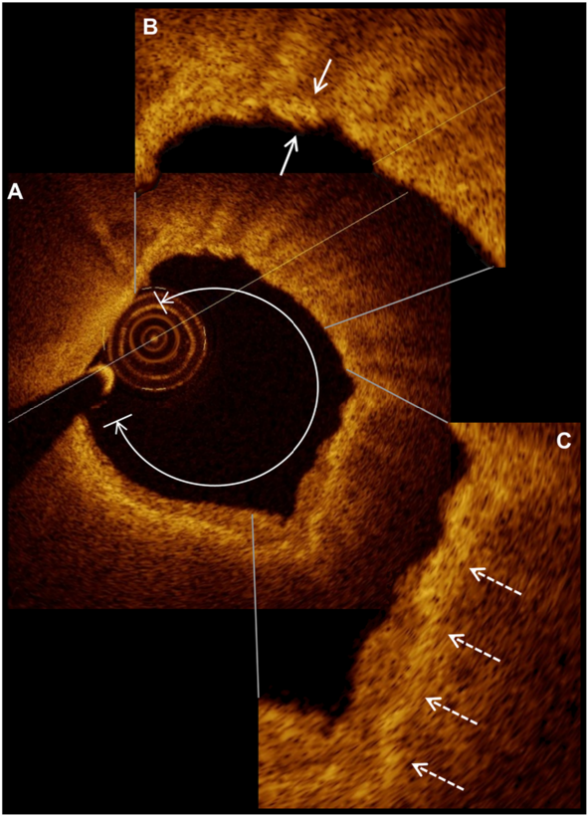
**Representative optical coherence tomography images.** A lipid-rich plaque **(A)** with a lipid arc of 282° is displayed. The right-upper high-power view **(B)** shows the fibrous cap measuring 52 μm (small arrows). Macrophages are visible in the right-lower high-power view **(C)** and are indicated with dashed arrows.

To evaluate both clinical and morphological plaque characteristics as risk factors for ACS, univariable logistic regression analysis has been performed and is depicted in Table 1. Several morphologic parameters were identified to be risk factors for ACS including minimal (OR per 10 μm 0.39, p < 0.001) and mean (OR per 10 μm 0.57, p = 0.001) FCT overlying a necrotic lipid core. Furthermore, parameters describing the lipid content of the lesion such as presence of lipid-rich plaques (OR 7.09, p < 0.001), mean lipid arc (OR per 90° 18.08, p < 0.001), lipid plaque length (OR 1.80, p < 0.001) as well as the lipid volume index (OR per 1000 1.53, p < 0.001) were significant risk factors that the lesion is the cause of an ACS. Further risk factors included the presence of macrophages (OR 8.75, p < 0.001) and microchannels (OR 2.30, p = 0.036). For further detail, please refer to Table 1, Figure 1 illustrates OCT-derived features of plaque vulnerability.

To investigate which of these risk factors predicted ACS independently, multivariable logistic regression analysis has been performed. As demonstrated in Table 2, only minimal FCT (OR per 10 μm 0.478, p = 0.002), mean lipid arc (OR per 90° 13.997, p < 0.001) and presence of macrophages (OR 4.797, p = 0.015) were independent predictors for the lesion to be the cause of an ACS. The regression model included also lipid plaque length (OR 1.290), which was not excluded from the equation due to p = 0.098.

**Table 2:** **Multivariable logistic regression analysis for the lesion to be the cause of an ACS**

**Parameter**	**Odds ratio**	**95% CI**	**p-value**
Minimal FCT (10 μm)	0.478	0.302 - 0.757	0.002
Macrophages (present)	4.797	1.351 - 17.033	0.015
Lipid arc (90°)	13.997	3.186 - 61.494	<0.001
Lipid plaque length (mm)	1.290	0.954 - 1.745	0.098

Abbreviations as in Table 1.

### Diagnostic efficiency to predict plaque vulnerability

**Figure 2:**
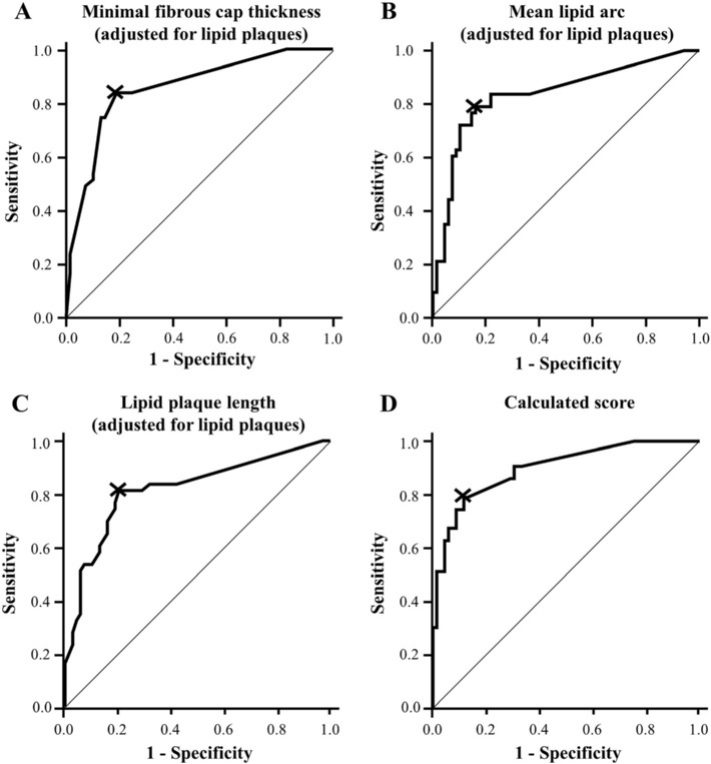
**OCT-derived parameters identify lesions of patients with ACS both individually and when combined in a score.** ROC curve for OCT-derived **(A)** minimal fibrous cap thickness, **(B)** the mean lipid arc and **(C)** the lipid plaque length to identify lesions of patients with ACS both individually and when combined to a score **(D)**. **A-C** were adjusted for the presence of lipid plaques. The score also including the presence of macrophages is calculated as described in the text.

In order to evaluate the diagnostic efficiency of OCT-derived parameters to identify culprit lesions of patients with ACS, ROC statistics has been performed (Figure 2, Table 3).

**Table 3:** **ROC-analysis: OCT-derived parameters identify culprit lesions of patients with ACS**

	**AUC (95% CI)**	**Cut-off**	**Sens.**	**Spec.**	**PPV**	**NPV**
Macrophages		Present	81.4%	66.7%	60.3%	85.2%
Minimal FCT*LP	0.86 (0.78-0.93)	74.5 μm§	83.7%	81.2%	73.5%	88.9%
Lipid arc*LP	0.84 (0.76-0.92)	116.73°§	79.1%	84.1%	75.6%	86.6%
Lipid plaque length*LP	0.82 (0.73-0.91)	3.10 mm§	81.4%	79.7%	71.4%	87.3%
Score	0.90 (0.84-0.96)	0.336	79.1%	88.4%	81.0%	87.1%

Abbreviations as in Table 2. *AUC* area under the curve, *Sens* Sensitivity, *Spec* Specificity, *CI* confidence interval, *PPV* positive predictive value, *NPV* negative predictive value, *LP* lipid plaque, § or non-lipid plaque.

Among all independent risk factors for ACS, minimal FCT (adjusted for the presence of lipid-rich plaques) had the best diagnostic efficiency (AUC 0.86, optimal cut-off value 74.5μm (or non-lipid plaque), sensitivity 83.7%, specificity 81.2%, PPV 73.5%, NPV 88.9%).

However, if all four independent parameters were combined to a score using the equation derived from the multivariable logistic regression model (Logit(Score) = -2.401 + 1.568 * (insert 1 if macrophages present; else 0) + 2.639 * (insert medium lipid arc in multiples of 90°) + 0.255 * (insert lipid plaque length in mm) - 0.738 * (insert minimal FCT in multiples of 10 μm), the diagnostic efficiency could be improved to an AUC 0.90 (95% CI 0.84-0.96, optimal cut-off value 0.336, sensitivity 79.1%, specificity 88.4%, PPV 81.0%, NPV 87.1%).

**Figure 3:**
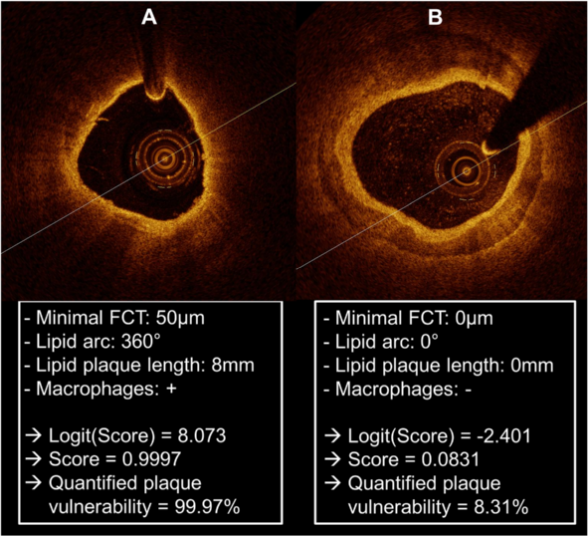
**Examples for the quantification of lesion vulnerability.** Representative images of a vulnerable lipid-rich **(A)** and a stable calcified **(B)** intracoronary plaque as derived from optical coherence tomography are presented with values for the minimal fibrous cap thickness (FCT), the lipid arc, the lipid plaque length and the presence of macrophages. The score is calculated in these two examples and the absolute probability of the plaque to be the cause of an ACS (≈quantified plaque vulnerability) is presented.

For illustration, this score is used in Figure 3 to quantify the vulnerability of two lesions.

## Discussion

The main findings of this study in patients with type 2 diabetes are:

1. Whereas several OCT-derived plaque parameters are predictors for ACS, only mean lipid arc, lipid plaque length, presence of macrophages and minimal FCT were independent predictors.
2. When combined to a score, these four independent risk factors identify culprit lesions of patients with ACS with excellent diagnostic efficiency.

### Features of the ruptured plaque

Several investigations have described certain lesion morphologies to be found more frequently in patients with ACS compared to those with SAP [2-4,6]. Thus, histopathological studies have identified a lower FCT (<65 μm) overlying a large lipid core, a higher incidence of thin-capped fibroatheromas (TCFA) and an increased macrophage infiltration within the plaque [2,15,16] as pivotal precursors for plaque rupture and concomitant cardiovascular events in ACS patients [17].

With the emerging use of virtual histology IVUS it was possible to prospectively confirm that ACS usually arise from the rupture of insignificant lesions with a thin FCT [10]. The Providing Regional Observations to Study Predictors of Events in the Coronary Tree (PROSPECT)-study demonstrated that coronary non-culprit lesions causing future major adverse cardiovascular events had a baseline diameter stenosis of only 32%, but were associated with more TCFAs, a greater plaque burden and a minimal lumen area <4 mm^2^ using virtual histology IVUS [10]. On the contrary, when focusing on coronary culprit lesions, the above and several other studies indicated that lesion severity in culprit lesions is generally greater than 50% in the majority of ACS cases [10,18-20]. In this context, Tian et al. compared morphologic plaque characteristics between culprit and non-culprit lesions and reported that plaque rupture is determined by FCT and a combination of large plaque burden and luminal narrowing is a prerequisite for the development of acute coronary syndromes [19]. These findings are in line with the ATHEROREMO-IVUS study, which recently identified TCFAs and a large plaque burden (>70%) as independent predictors for the occurrence of cardiovascular events in coronary non-culprit lesions [21]. The Clinical Outcomes Utilizing Revascularization and Aggressive Drug Evaluation (COURAGE) study revealed that anatomic burden of coronary artery disease predicts the risk of death, myocardial infarction and non-ST-elevation ACS [22].

Furthermore, subjects with extensive coronary artery calcification as derived by computed-tomography were associated with a rather stable clinical course and presented a high level of chronic cardiovascular events compared to patients with mild and moderate coronary artery calcification, which more frequently presented with an ACS [23]. This finding strengthens our observations in the present study as it indicates a potentially plaque-stabilizing effect of coronary calcification, whereas soft, predominantly lipid-rich lesions with a thin fibrous cap may rather tend to rupture due to their lower resistance towards intracoronary forces [23].

Currently there is a paucity of data regarding vulnerable plaque criteria in patients with type 2 diabetes. However, diabetic patients are at a particular high risk for cardiovascular events and the identification of vulnerable plaques and vulnerable plaque features is crucial in this population. Our own group has previously demonstrated that diabetic patients with ACS exhibit more vulnerable plaque characteristics in coronary target lesions compared to diabetics with SAP [5]. Moreover, we were able to point out an association between a lower minimal fibrous cap thickness of coronary culprit lesions to both hemodynamic relevance of a coronary lesion as well as to left ventricular dilatation in exclusively diabetic cohorts [12,24].

The present study is consistent with the above investigations and adds to the current knowledge by the identification of vulnerable plaque features in patients with type 2 diabetes. Specifically, we demonstrate more frequent lipid-rich plaques, a larger necrotic core and a lower fibrous cap thickness in diabetic patients with ACS compared to those with SAP.

### Quantifying plaque vulnerability

However, the individual and combined power of these plaque features to quantify plaque vulnerability is incompletely understood. To the best of our knowledge this study is first to summarize these previously and individually established parameters of plaque vulnerability to a score. Whereas we and others have found non-plaque derived parameters such as left ventricular dilatation to be predictors for plaque vulnerability [12,24,25], we confined the present investigation to OCT-derived plaque parameters to quantify plaque vulnerability. Using the novel imaging modality OCT with its superior resolution compared to IVUS, the present analysis was able to include additional parameters of plaque vulnerability, such as macrophage infiltration and the presence of microchannels [7,8]. Thus, we expand the current knowledge by demonstrating that among all morphologic plaque parameters studied by OCT, only minimal FCT, presence of macrophages, mean lipid arc and lipid plaque length are independent predictors of the lesion to be the cause for an ACS in patients with type 2 diabetes. Whereas previous studies have also described these parameters as vulnerable plaque features [10,26], we found that minimal FCT had the best diagnostic efficiency to quantify plaque vulnerability among all OCT-derived parameters investigated.

Furthermore, when combined to a score, these risk factors offer an excellent diagnostic efficiency to quantify plaque vulnerability and to identify culprit lesions of diabetic patients with ACS.

### Using plaque vulnerability to guide coronary interventions

The Fractional Flow Reserve Versus Angiography for Multivessel Evaluation (FAME)-study had previously demonstrated a reduction in cardiovascular events if coronary interventions were guided by fractional flow reserve (FFR) rather than angiography alone [27,28]. However, neither ischemic nor anatomic burden were able to distinctly identify patients who would benefit from an invasive revascularization strategy over optimal medical therapy in COURAGE [22]. Thus, these data emphasize the importance of optimal medical therapy on the one hand and also – more importantly – the need for alternative diagnostic and therapeutic strategies to guide and optimize coronary interventions on the other hand. In this regard, a score to quantify the vulnerability of a coronary lesion might be of interest.

Using the OCT-risk score for plaque vulnerability presented in this study it may be possible to unmask those lesions at risk to cause an ACS in high-risk patients with type 2 diabetes. Therefore, our findings possibly indicate that OCT may be essential for guiding the clinical decision for revascularization beyond angiographic or hemodynamic lesion severity. As this novel score focuses on the overall and combined impact of single features of plaque vulnerability it may have the potential to be a valuable and convenient tool for clinical risk-stratification and clinical decision-making. Moreover, this score may support and extend the clinical indication spectrum for OCT and in selected cases may be an alternative and/or complement to FFR, which has hitherto been the gold-standard for evaluating the PCI indication [12,27,28]. This applies in particular to lesions in which the relevance of the FFR technique is either limited, such as in case of serial stenoses or challenging anatomic sites, like left main or bifurcation lesions, or if FFR measurements lack clear hemodynamic significance despite advanced angiographic stenosis severity or persistent clinical symptoms.

Thus, this score may help to guide PCI in lesions in which FFR measurements are either not reliable or applicable. However, if FFR assessment displays hemodynamic relevance of a coronary stenosis, PCI should be performed irrespective of the value of our score.

Future studies are warranted to evaluate this score in prospective trials and to determine if coronary interventions guided by this score reduce cardiovascular morbidity and mortality in patients with type 2 diabetes. The present investigation may be the basis for further clinical studies to validate this score in larger diabetic as well as non-diabetic cohorts.

### Limitations

Although the current study is hitherto the largest OCT-study in an exclusively diabetic cohort, the relatively small sample size limits the precision of the regression coefficients.

Second, large randomized studies are required to evaluate whether our score may actually help to reduce cardiovascular morbidity and mortality in high-risk patients with type 2 diabetes.

Third, as we have excluded patients with hemodynamic instability or cardiogenic shock our data may only partially reflect the true incidence of plaque ruptures and specific plaque features in ACS patients.

Finally, as this is a study in an exclusively diabetic cohort, future studies are needed to determine if this score can also be applied to patients without diabetes.

## Conclusion

According to the findings of this OCT-study we conclude that in patients with type 2 diabetes minimal FCT, presence of macrophages, mean lipid arc and lipid plaque length are independent morphologic predictors for acute coronary events. When combined to a score these parameters have an excellent diagnostic efficiency to quantify plaque vulnerability. Future interventional studies are warranted to determine if this score may help to reduce cardiovascular morbidity and mortality in high-risk patients with type 2 diabetes.

## Abbreviations

ACS: Acute coronary syndrome
IVUS: Intravascular ultrasound
OCT: Optical coherence tomography
FCT: Fibrous cap thickness
SAP: Stable angina pectoris
QCA: Quantitative coronary angiography
OR: Odds ratio
CI: Confidence interval
PPV: Positive predictive value
NPV: Negative predictive value
ROC: Receiver-operated curve
AUC: Area under the curve
TCFA: Thin-capped fibroatheroma
